# Ubiquitin-specific protease 7 is a drug-able target that promotes hepatocellular carcinoma and chemoresistance

**DOI:** 10.1186/s12935-020-1109-2

**Published:** 2020-01-28

**Authors:** Wei Zhang, Jingxin Zhang, Chenzhou Xu, Shiqing Zhang, Saiyan Bian, Feng Jiang, Wenkai Ni, Lishuai Qu, Cuihua Lu, Runzhou Ni, Yihui Fan, Mingbing Xiao, Jinxia Liu

**Affiliations:** 1 Department of Gastroenterology, Affiliated Hospital of Nantong University, Nantong, 226001 Jiangsu People’s Republic of China; 2 Medical College, Nantong University, Nantong, 226001 China; 3 Laboratory of Medical Science, School of Medicine, Nantong University, Jiangsu, 226001 China; 4 Research Center of Clinical Medicine, Affiliated Hospital of Nantong University, Nantong, 226001 Jiangsu People’s Republic of China

**Keywords:** Hepatocellular carcinoma, Ubiquitin-specific protease 7, P22077, Apoptosis, Migration

## Abstract

**Background:**

Ubiquitin-specific protease 7 (USP7) is a de-ubiquitin enzyme that plays an essential role in multiple cancers and becomes a target for treatment. However, the role of USP7 and its therapeutic value for HCC remains unclear.

**Methods:**

USP7 expression was examined in HCC tissues by western blot and immunohistochemistry. The correlation of USP7 and HCC prognosis was analyzed by Kaplan–Meier survival method. Mass spectrometry was determined and cell proliferation and tumorigenicity assays were conducted in vitro and in vivo treated by P22077 and sgRNA-USP7.

**Results:**

USP7 expression was significantly increased in HCC and associated with its progression. Interestingly, many HCC cells are sensitive to USP7 inhibition by using P22077. P22077 treatment not only induced cell death but also inhibited cell proliferation and migration in Huh7 and SK-Hep1 cells. In a xenograft model, P22077 efficiently inhibited tumor growth. In chemo-resistant HCC cells, P22077 decreased cell sensitivity to chemotherapy. In addition, mass spectrometry reveals 224 of significantly changed proteins upon P22077 treatment.

**Conclusions:**

We demonstrate a critical role of USP7 in HCC devolvement and chemoresistance. Disruption of USP7 function results in dis-regulated several key biological processes and subsequently activates BAX. USP7 might be a novel and drug-able target in HCC.

## Background

HCC is one of the most common malignant tumors in the world, and its mortality has increased year by year [1]. Deaths caused by HCC worldwide has increased at a greater rate compared to all other cancer [2]. The advanced cancer-stage patients were treated with chemotherapy or radiation which can be used along with surgery. However, these therapeutic protocols frequently result in the occurrence of drug resistance in the cancer cells to the treatment [3, 4]. Therefore, it is particularly important to identify more efficient treatment for HCC.

Deubiquitinase is involved in the regulation of cell cycle progression, signal transduction pathway regulation, gene expression, DNA damage repair and chromosome segregation [5, 6]. USP7 is a member of the deubiquitinating enzymes families and contains five consecutive ubiquitin-like regions (UBLs) [7]. USP7 was involved in host-virus interaction, DNA damage and repair, gene expression and protein function regulation, immunization and others cellular process [8]. USP7 plays a multifaceted role and is involved in the development of a variety of tumors, including esophagus cancer, myeloma and ovarian cancer [9–11]. USP7 also regulated the expression of a variety of tumor suppressor genes. In cancer, USP7 could stabilize MDM2 by de-ubiquitination and subsequently promotes degradation of P53 specifically [12]. Moreover, USP7 impacts mainly on the nuclear activities of PTEN and FOXO, which were forced to the nucleus by USP7 inhibition and sufficient to induce cancer cells apoptosis [13, 14]. In HCC, USP7 was highly expressed in cancer tissues [15]. USP7 could inhibit the ubiquitination of P65 and stabilize the expression of P65, thereby promoting the progress of hepatoma cancer [16]. In addition, reported data supports that USP7 can be used as a new independent prognostic factor for liver cancer [17]. However, compared to extensive studies regarding USP7 in several other cancers, the function of USP7 in HCC remains to be fully addressed.

In recent years, due to the critical role of USP7, its inhibitors have been interested for cancer treatment. Earlier studies showed that the small molecule compounds HBX 41,108 were regulated the expression of P53 by inhibiting the de-ubiquitination activity of USP7 and suppressed the growth of cancer cells [18]. The USP7 inhibitor P5091 inhibits Ewing’s sarcoma growth and extends survival time by combined with other treatments [19]. Recently, a novel small molecule (P22077) that specifically inhibits the function of USP7 has been developed. In vitro assays indicate that P22077 showed low dose inhibitory activity against cancer cell lines via a p53-dependent and p53-independent mechanism [20]. In addition, not only the tumor cell growth was significantly delayed after knocking down USP7, but also the cellular toxicity of paclitaxel and docetaxel but not carboplatin and cisplatin was enhanced significantly [21]. However, it is still unknown whether P22077 has therapeutic potential in the treatment of HCC. Here, we revealed an important role of USP7 in HCC and targeting USP7 might be an efficient way to kill HCC cells. The USP7 inhibitor also has synergetic effect with chemotherapeutic drug in the killing of HCC.

## Materials and methods

### HCC tissue and cells

The HCC fresh tissue from HCC patients (Affiliated hospital of Nantong University). The liver cell (LO2) and HCC cells (SMMC-7721, SK-Hep1, HepG2, Huh7) were purchased from ATCC (USA). LO2 was cultured in 1640 DMEM + 10% FBS. HCC cells were cultured in DMEM + 10% FBS. All cells lines were cultured in 37 °C with 5% CO2 concentration.

### Immunohistochemistry (IHC) and evaluation

The paraffin-embedded tissues sections from 100 matched pairs of liver adenocarcinoma and nonmalignant liver tissue of the same patients. The tissues were heated at 60 °C for 45 min and deparaffinized using a graded ethanol series. Then, these tissue sections were incubated in sodium citrate buffer and heated for antigen retrieval and endogenous peroxidase activity was blocked by soaking in 0.3% hydrogen peroxide. USP7 (Santa Cruz, CA, USA) was incubated for 1 h at room temperature. The second antibody was then incubated. All sections were counterstained with hematoxylin, dehydrated and evaluated. Staining of USP7 in the tissues was reviewed and scored independently by two pathologists blinded to the clinical data. The intensity of immunostaining was documented as 0–3 (0, negative; 1, weak; 2, moderate; 3, strong). The percentage of immunoreactive cells was documented as 1 (0–25%), 2 (26–50%), 3 (51–75%), and 4 (76–100%). Under these conditions, the optimum cut-off value of samples were respectively classified as low and high expression USP7 by receiver operating curve (ROC) analysis. The area under the curve (AUCs) at different cut-off values of USP7 for different years of overall survival time were calculated.

### Antibodies and reagents

The USP7 inhibitor P22077 and Doxorubicin (Dox) was purchased from Selleckchem (Houston, TX, USA), dissolved and diluted in dimethyl sulfoxide (DMSO; Sigma, St. Louis, MO, USA), and stored at − 20 °C, DOX was keep from light. The antibodies were used in Western Blot: anti-USP7 (Santa Cruz Biotechnology, Santa Cruz, CA, USA), anti-H3, anti-H3K4me^2^, anti-Bax, anti-GAPDH (Cell Signaling Technology, Inc, Beverly, MA, USA).

### Western blot analysis

The whole cell lysates come from the cell which were treatment with USP7 inhibitor for 48 h at the indicated doses, and was measured using western blot analysis. The protein were subjected to SDS-PAGE and transferred to PVDF membranes (New England Nuclear, Boston, MA,USA). Subsequently,the PVDF was membranes was blocked with 5% nonfat dry milk with TBS-T which containing Tris-buffered saline and 0.1% Tween-20. Probed with the primary antibodies. After washing with them TBS-T buffer, the cell lysates were incubated with the secondary antibody. The chemiluminescence system (Bio-Rad, USA) was used to detect the protein expression.

### CRISPR- sgRNA construction

We designed two sgRNA to knock out the potential sequence of USP7. The vector plasmids were digested by BSPQI enzyme (New England Biolads) at 50 °C for one hour. The sense and antisense sgRNA oligos targeting the predicted super-enhancer locus were annealed at 90 °C for 20 min followed by cooling at room temperature for an hour. The annealing buffer was as following: 10 mM Tris (pH 7.5), 1 mM EDTA and 50 mM NaCl. The ligation was executed with T4 DNA ligase (New England Biolads) at 16 °C overnight. All plasmids were validated by using enzyme digestion and direct DNA sequencing.

### Cell transfection

Place the cells into a 6-well plate at the appropriate density. After the cells adhere to the cells within 24 h, transfection is performed. Lipofectamine 2000 was diluted in DMEM medium at various concentrations to find the optimal transfection dose per cell. Mix appropriate amounts of DNA and Lipofectamine 2000 with a little DMEM, respectively, and incubate for 5 min at room temperature. Finally, the DNA was mixed with Lipofectamine 2000 and incubated for 15 min, then added to the cell culture medium, and the medium was changed 6 h after transfection. Due to the high transfection efficiency of the HEK293T cell line, all of our SgRNA transfection was performed first in HEK293T cells. After confirming its knockout in HEK293T cells, stable cell lines with corresponding knockouts will be established in tumor cells.

### Establishment of stable cell lines

To construct stable USP7 knockout cell line, SK-hep1 cells and Huh7 cells were transfected by SgUSP7-1, SgUSP7-2 plasmids and transfected by epi-CRISPR vector plasmid as control. After 48 h of transfection, the medium was changed by complete medium containing 1 μg/ml puromycin (InvivoGen). Puromycin-containing medium was further used for additional 2 weeks to select the resistant cells until the stable cell line was established, and always use a concentration of 0.5 μg/ml puromycin to maintain.

### Cellular immunofluorescence assays

Suitable cells were in 24-well plate treated with P22077 for 12 h, subsequently, cells treated with Dox at indicated doses for 48 h. Cells were fixed by 4% Paraformaldehyde after the cells rinsed by PBS for 3 times. Cells were closed with Triton X-100 for 30 min and then using anti-USP7 antibody at overnight. After washing with the PBS, the cells were stained with fluorescent dyes (Themorfer, USA) for 2 h and take photos from the light.

### CCK-8 assays

The cell proliferation and viability were detected by the cell counting kit-8 (CCK-8; Best Bio, Shanghai, China) assay. Cells were inoculated into 96-well plates at a density of 2 × 10^4^/well and cultivated for 24 h. Each well incubated with CCK-8 reagents for another at the same time on consecutive days. Last, the automated plate reader was used to read the absorbance at a wavelength of 450 nm.

### Flow cytometric assays

Cells were treated with P22077 or Dox for 48 h were prepared for the apoptosis detection assay. Cells were washed in PBS at least three times, resuspended in 195 μl of binding buffer and incubated with Annexin V-fluorescein isothiocyanate (Best Bio, Shanghai, China) for 15 min in the dark. By followed, cells were incubated with PI (Best Bio, Shanghai, China) in the dark for 5 min, then analysed by flow cytometry (Beckman Coulter Inc., USA) processes.

### Colony formation assays

500–1000 cells in a six-well plate were incubated for 24 h and then p22077 were added to stimulating the cells for 2 weeks. Colonies which were formed macroscopic, fixed by 4% Paraformaldehyde for 2 h and the purple crystal were used to color. The colonies were counted and calculated compared with the control group.

### Wound healing assay

The Scratch assays was used to examined the migration of the cell treated with P22077 at indicated doses for 24 h or 48 h. Subconfluent cells of each group were scraped using sterilized 10 µl pipette tips, washed with PBS, and cultured in six-plated. Wound healing was observed under the microscope (USA) and capture the images.

### Allograft assay

Four week old male nude mice were purchased from Nantong University Animal Center (Nantong, China) and the SK-Hep1 cell were injected in specific pathogen-free conditions for 5 × 10^6^ cells into left flank of the nude mice. The nude mice were divided into two groups contained the control group and the experimental group with three mice. P22077 was injected into mice at the dose of 10 mg/kg by intraperitoneal injection of the experimental group. All mice were sacrificed after 6 weeks. Tumors were removed, weighed, and fixed in formalin for the subsequent analysis.

### Statistical analysis

All statistical analyses were carried out using GraphPad version 5.0 (Graph Pad software, USA). Significance was analyzed using SPSS 15.0 and student’s t-test. All data are shown as means and standard errors of the mean. P < 0.05 was considered indicate a statistically significant.

## Results

### USP7 overexpression correlates with HCC progression

**Fig. 1:**
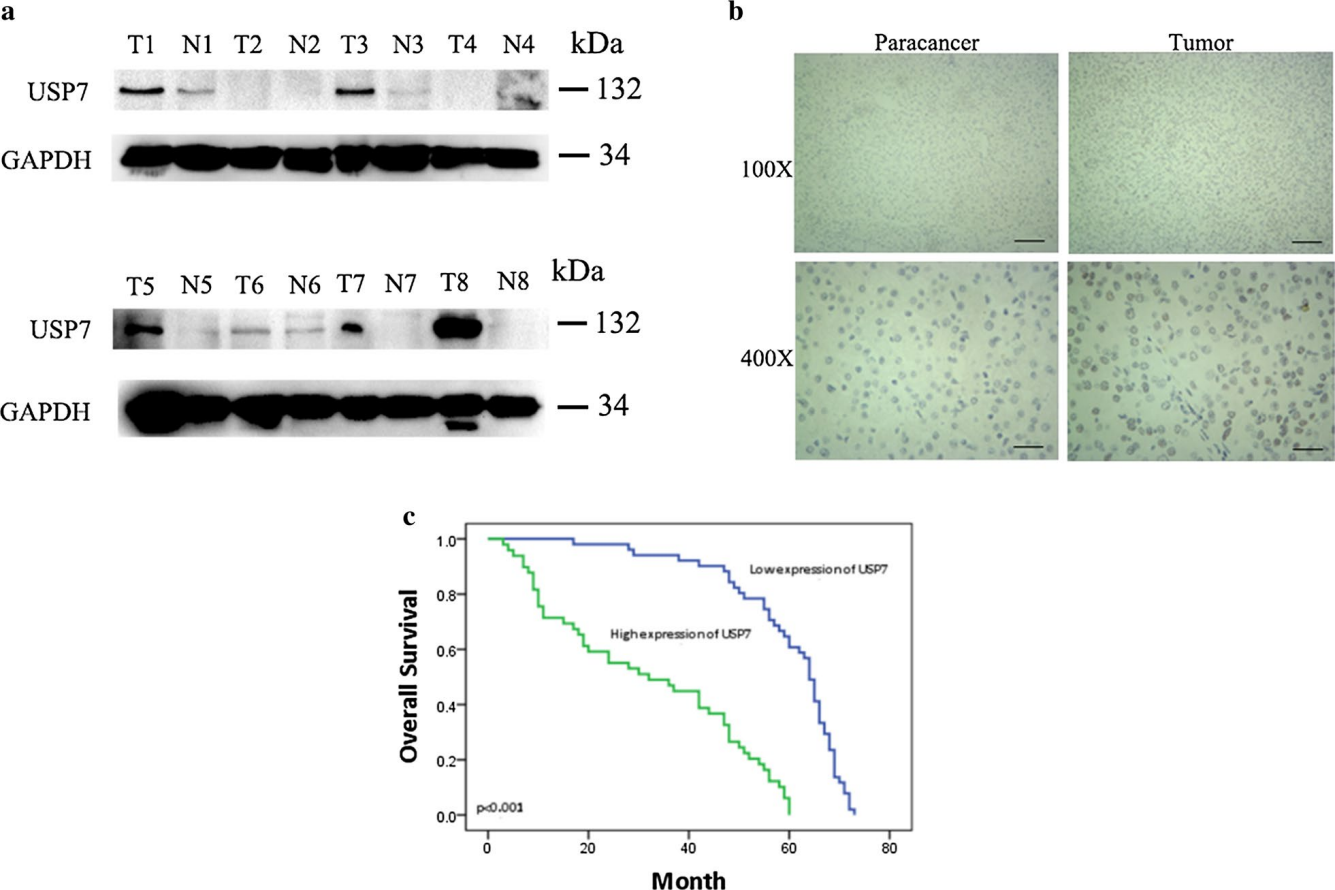
USP7 was up-regulated in HCC tissues and associated with patients’ outcome. **a** The expression of USP7 was detected by Western Blot in HCC tissues (T) and its non-cancerous adjacent tissues (N). **b** USP7 was highly expressed in the nuclear of tumor tissues compare with normal tissues by immunohistochemistry. Representative examples of USP7 staining were shown; Scale bars: 100 μm (×100), 25 μm (×400). **c** Kaplan–Meier survival analysis showed that patients with higher expression of USP7 had short overall survival times

USP7 acts as a deubiquitinating enzyme not only contributes to tumorigenesis but also plays critical role in response to therapy. It becomes one of most interesting drug-able targets in cancers but its role as well as potential therapeutic value in HCC remains unclear. We firstly explored USP7 expression using WB in 8 paired HCC tissues. In majority of cases, USP7 was overexpressed in most of HCC tissues than adjacent tissues (Fig. 1a). To further confirm this finding, we examined the expression and distribution of USP7 in HCC tissues by IHC. As showed in Fig. 1b, adjacent liver tissues showed negative staining or slight positive staining, while HCC tissues possess large amount of USP7 and USP7 predominantly locates in the nucleus (Fig. 1b). In the cohort of 100 HCC patients, USP7 expression is highly correlated with unfavorable clinical outcomes (Fig. 1c). Together, our results clearly demonstrate that USP7 is correlated with patient outcome. To further explore the role of USP7 in HCC development, we analyzed which factors are associated with the level of USP7. We divided HCC patients into different groups based on the IHC scores of USP7. As shown in Table 1, high USP7 level was associated with tumor size (*P *= 0.018), tumor differentiation (*P *= 0.007), advanced TNM stage (*P *= 0.004) and liver cirrhosis (*P *= 0.025). These results indicates that USP7 plays a critical role in the development of HCC and its expression is associated with patients’ outcome.

**Table 1:** Association of USP7 expression with clinicopathological parameters in 100 HCC patients

Parameters	Total	USP7 expression		*P* value
		High	Low	
Sex				0.362
Male	82	39	43	
Female	18	10	8	
Age (years)				0.487
< 50	42	20	22	
≥ 50	58	29	29	
AFP (ng/ml)				0.556
< 400	67	33	34	
≥ 400	33	16	17	
Tumor size (cm)				0.018*
< 5	40	14	26	
≥ 5	60	35	25	
Tumor number				0.247
Single	82	42	40	
Multiple	18	7	11	
Tumor differentiation				0.007*
I-II	75	31	44	
III	25	18	7	
Portal vein tumor thrombus				0.149
Yes	25	15	10	
No	75	34	41	
TNM stage				0.004*
I-II	34	10	24	
III-IV	66	39	27	
HBV infection				0.548
Yes	76	37	39	
No	24	12	12	
Liver cirrhosis				0.025*
Yes	65	37	28	
No	35	12	23	

*HCC* hepatocellular carcinoma, *AFP* α-fetoprotein, *HBV* hepatitis B virus, *TNM* topography, lymph node, metastasis. Statistical analyses were performed by the Pearson χ^2^ test

* P < 0.05 was considered significant

### USP7 is required for survival in HCC cells

**Fig. 2:**
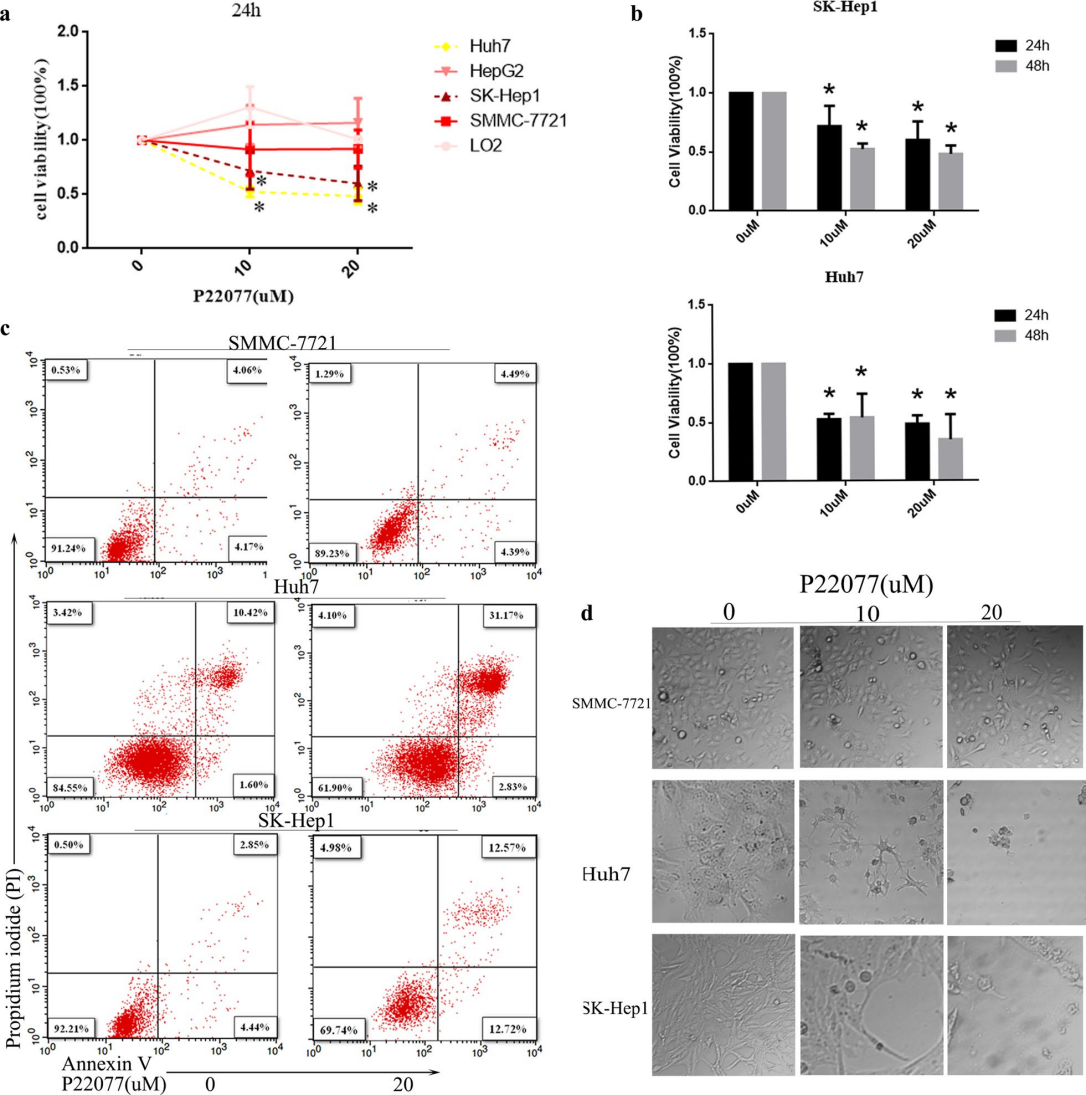
Liver cancer cells suffer both necrosis and apoptosis following P22077 treatment. **a**, **b** The doses of P22077 was indicated and cell viability was measured with CCK-8 assays in LO2, SMMC-7721, SK-Hep1, HepG2 and Huh7 cells for 24 h or 48 h. **b** data were presented as mean ± SD. **c** Three HCC cell lines including SMMC-7721, Huh7 and SK-Hep1 were treated with P22077 for 24 h and stained with PI and Annexin V. Cells were analyzed using a flow cytometry. **d** Treatment of HCC cells with 10 µM or 20 µM P22077 for 24 h resulted in a significant death of cells as determined by phase contrast microscopy. All *P < 0.05 was considered significant

Due to the high expression of USP7 in HCC and the correlation between USP7 and patients outcome, we examined whether HCC cells relies USP7 for survival. The USP7 inhibitor P22077 was used to treat a panel of HCC cell lines (Huh7, HepG2, SK-Hep1, SMMC-7721) and control cells (LO2). After treatment for 24 h, the viability of Huh7 and SK-Hep1 is significantly reduced under the dose of 10 or 20 µM. Under the same condition, P22077 has minimal effect on control cells (LO2) as well as HCC cells (HepG2, SMMC-7721) (Fig. 2a). Increased treatment time further induces cell death in HuH7 cells and SK-Hep1 cells (Fig. 2b). The same results were also confirmed in USP7 deficient stable cell lines including HuH7 and SK-Hep1 in Additional file 1: Figure S1c. Flowcytometry analysis demonstrated that P22077 treatment induces apoptosis in Huh7 and SK-Hep1 cells but not in SMMC-7721 cells (Fig. 2c). We chose SK-Hep1 USP7 stable cells to reach the same conclusion (Additional file 1: Figure S1e). Microscopic images of cell morphology further showed that P22077 dramatically induced cell death in Huh7 and SK-Hep1 cells but not in SMMC-7721 cells (Fig. 2d). Consistently, we found that the function of USP7 is essential of partial of HCC cells.

### Effect of P22077 on colony formation and migration

**Fig. 3:**
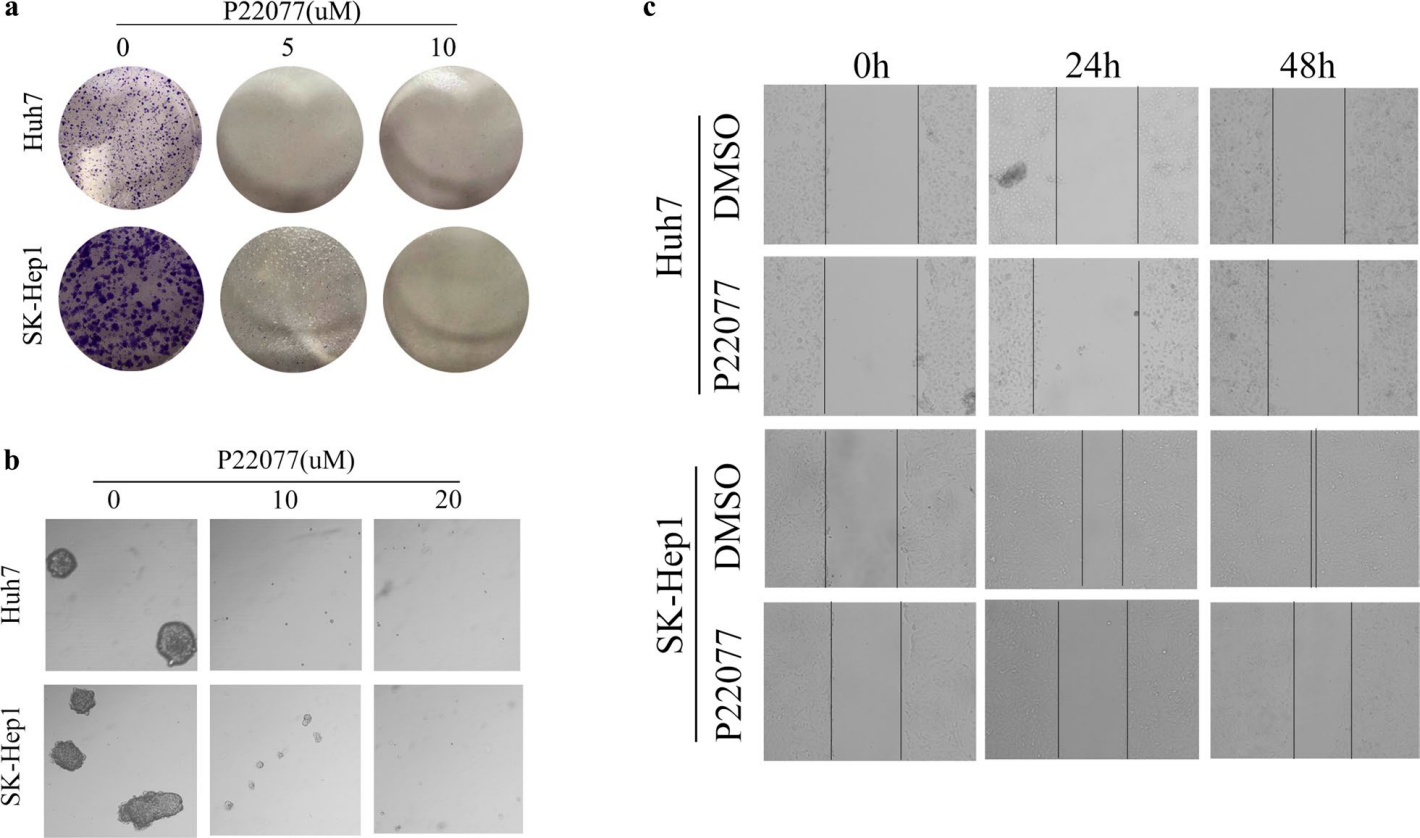
A dose-dependent effect of P22077 on the colony-forming ability and migration of HCC cells. **a** HuH7 and SK-Hep1 cells were treated with or without P22077 for 2 weeks. The number of colonies were counted and stained with crystal violet. **b** Anchor-dependent colony formation was measured with or without P22077 treatment. **c** The effect of P22077 on cell migration was examined by wound healing assay

We further verify the potential anti-tumor ability of P22077 on HCC cells, Huh7 and SK-Hep1 were treated with P22077 at a concentration of 5 µM, 10 µM and 20 µM. As shown in Fig. 3a, the number of cell foci were obviously decreased in lower dose, the higher dose almost completely abolished the anchor dependent colony-forming ability of these two cells. The similar result was shown in Fig. 2b, P22077 drastically reduced ability of anchor-independent colony formation in Huh7 and SK-Hep1 (Fig. 3b). Subsequently, in vitro scratch assays were undertaken. Compared with DMSO group, Huh7 and SK-Hep1 exhibited delayed wound healing after treated with P22077 for 24 h or 48 h (Fig. 3c). Colony formation, anchor-independent colony formation and scratch assays are also verified in USP7 deficient stable cells including SK-Hep1 and Huh7 (Additional file 1: Figure S1 a, b, f). Taken together, USP7 is required for HCC colony formation and migration.

### P22077 inhibits the growth of HCC in vivo

**Fig. 4:**
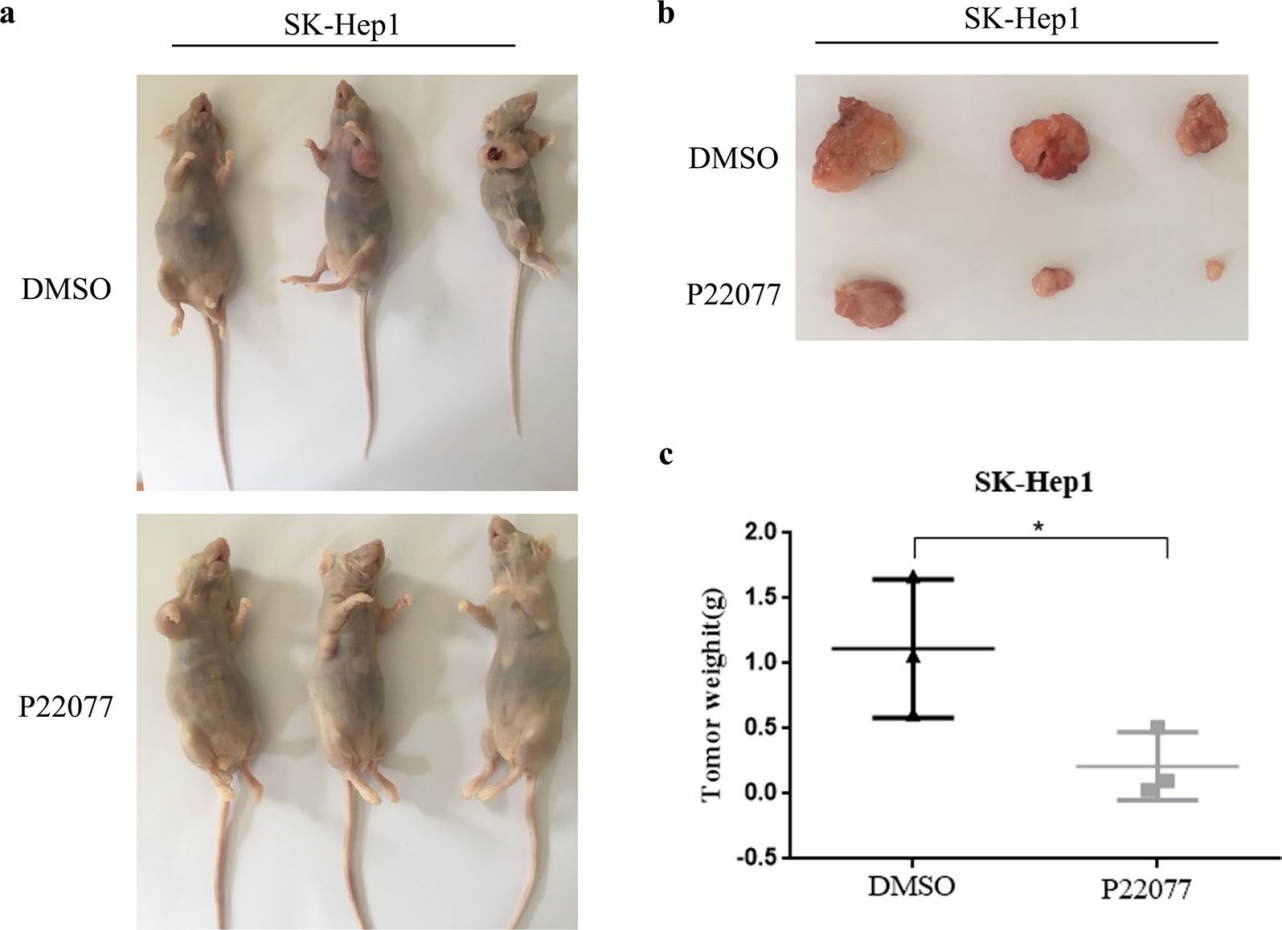
P22077 inhibits HCC growth in xenograft mice. **a** P22077 inhibited growth of SK-Hep1 xenograft tumors. Xenograft tumors were generated by injecting SK-Hep1 cells with or without P22077 at dose of 10 mg/kg. **b**, **c**, the growth of xenograft tumors was moved and measured, the tumor weight was recorded. All *P < 0.05 was considered significant

Next, we examined whether P22077 could inhibits the growth of HCC in vivo. Hepatoma cells SK-Hep1 were injected subcutaneously in nude mice with concentration of 5 × 10^6^/cell. We divided the nude mice into two groups and treated them with DMSO and P22077, respectively. P22077 was injected intraperitoneally at 10 mg/kg for once every 2 weeks. All mice were sacrificed after 6 weeks, the tumors were weighted and photographed. Compared with DMSO-treated group, P22077 significantly inhibited the tumor growth (Fig. 4a, b). The weight of tumor mass in P22077-treated group was significantly decreased compared to control group (Fig. 4c). Our results revealed that P22077 is a potential anti-tumor drug for significantly inhibiting tumor growth.

### P22077 promotes the cellular toxicity of Dox on HCC cells

**Fig. 5:**
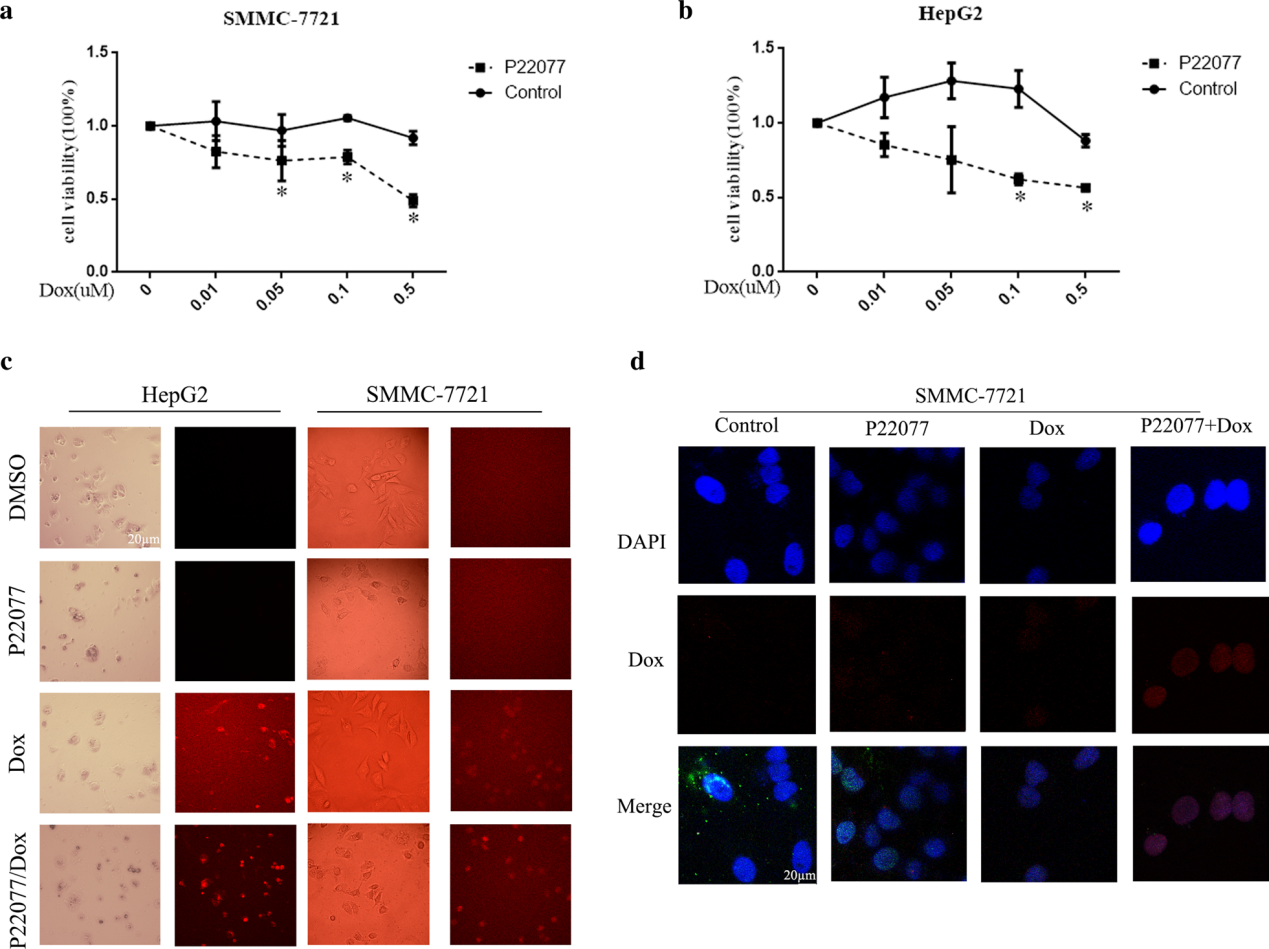
P22077 enhances the cellular toxicity of Dox in HCC cells. **a**, **b**, SMMC-7721 and HepG2 cells were incubated with increasing concentrations of Dox (0, 0.01, 0.05, 0.1, and 0.5 µM) plus DMSO or 10 µM of P22077 for 48 h. The cell number was measured by a CCK-8 assay. **c** SMMC-7721 and HepG2 cells were treated with DMSO, P22077 (10 µM), Dox (0.5 µM) and their combination for 48 h. The cells were photographed. Dox is red fluorescent. **d** Immunofluorescence analysis of distribution of Dox (0.5 µM) in SMCC-7721 cell treated with or withoutP22077(10 µM) for 48 h. DAPI was labeled the nuclear DNA. All * *P *< 0.05 was considered significant

By considering the potential application of P22077 in HCC therapy, we tested whether P22077 enhances the killing effect of chemotherapy. Previous experiments have shown that the tested concentration of P22077 has no significant effect on SMMC-7721 and HepG2 cells (Fig. 5a, b). We tested whether P22077 can increase the toxicity of Dox in SMMC-7721 and HepG2. Interestingly, SMMC-7721 and HepG2 are resistant to Dox in our tested doses. However, in the presence of P22077, SMMC-7721 and HepG2 cells are responsive to Dox and show a dose-dependent death. Doxorubicin has inherent fluorescence and thus can be visualized in cells. Consistent with cellular toxicity, the number of Dox-positive cells was significantly increased in the combination with P22077 (Fig. 5c). It suggests that P22077 might help Dox to enter nucleus. To test this hypothesis, we monitored the distribution of Dox in a course of P22077 treatment. As shown in Fig. 5d, Dox early enter into nucleus with the help of P22077. Taken together, our results indicate critical role of USP7 in cancer development and chemoresistance.

### P22077 treatment affects multiple essential biological processes and activates pro-apoptotic protein BAX

**Fig. 6:**
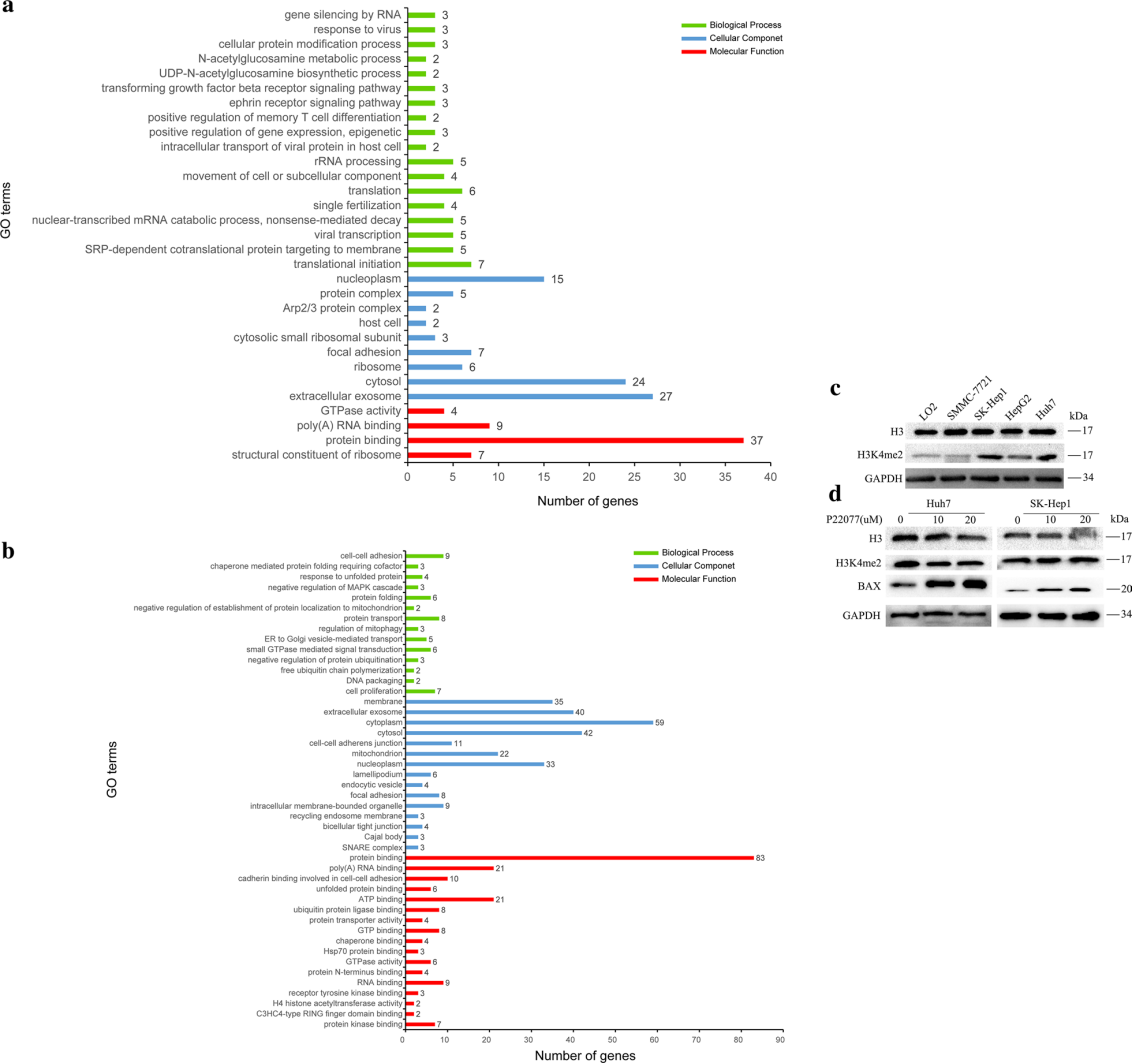
P22077 regulated multiple essential biological processes in Huh7 cell. Huh7 cells were treated with P22077 for 24 h. The treated and non-treated cells were harvested. Total protein was extracted and subjected for mass spectrometry analysis. **a** The identified down-regulated protein was further analyzed by pathway enrichment to identified significantly affected pathways. **b** The identified up-regulated protein after P22077 treatment was analyzed for pathway enrichment. **c** The expression of H3 and H3K4me^2^ was examined by Western Blot. **d** Cells were treated with P22077 and samples were resolved on SDS-PAGE gels and then transferred to nitrocellulose membranes for immunoblotting assays to detected the expression of H3, H3K4me^2^, BAX was probed to detect apoptosis. GAPDH was a loading control

Our results indicate that USP7 is a potential target for HCC treatment. To systematically understand the molecular mechanisms, we used mass spectrometry to globally analyze the protein change in response to P22077 treatment. In total, we identified 224 of significant changed proteins. Seventy-one of them are down-regulated while 153 of them are up-regulated (data not shown). Further analysis reveals that down-regulated proteins are enriched in several essential biological processes such as translational initiation, protein targeting to membrane, viral transcription, non-sense-mediated RNA decay (Fig. 6a). Up-regulated proteins are enriched in cell–cell adhesion, protein folding, protein transport, signal transduction and cell proliferation (Fig. 6b). This results suggest that USP7 is involved in many essential pathways in HCC. By looking at each down-regulated proteins, we interestingly found histone H3 is one of most affected protein after P22077 treatment. Due to the critical role of histone H3 in epigenetic regulation of transcription, we further confirmed the result obtained from mass spectrometry by WB. As shown in Fig. 6d, P22077 treatment slightly reduced the level of histone H3 in Huh7 and SK-Hep1 cells. The effect is much potent in the high dose of P22077. P22077 treatment also reduces the H3K4me^2^, which is a marker for transcriptional activation (Fig. 6d). Consistently, the P22077-sensative cell lines Huh7 and SK-Hep1 cells have relative high H3K4me^2^ compared to other tested cells (Fig. 6c). It might be partially explained why Huh7 and SK-Hep1 cells are more dependent on USP7 function. Disruption of the function of USP7 in Huh7 and SK-Hep1 cells greatly induces the expression of BAX, which is a potent inducer for apoptosis (Fig. 6d). Thus, P22077 treatment globally affects the biological processes in HCC and subsequent disruption of essential biological processes further induces BAX-mediated apoptosis.

## Discussion

Hepatocellular carcinoma (HCC) is one of the major malignancies in the world [22]. Surgical resection is still the main method of treating HCC [23]. Targeted therapy involving regorafenib, lenvatinib, cabozantinib and ramucirumab has also made great progress [24]. But the rapid development of HCC patients with poor prognosis and morbidity are increasing for years [25]. Therefore, it is urgent to find novel therapeutic strategies to improve patients’ survival.

Expression of deubiquitylase USP7 is reported to be a prognostic factor in osteogenic sarcoma, T-cell lymphoma and ovarian cancers [26–28]. Both the mRNA and protein expressions were obviously increased in HCC which is partly consistent with our results [29]. The higher expression of USP7 is correlated with poorer survival time. Datas indicated that the USP7 inhibitor P5091 was overcoming bortezomib resistance in multiple myelomas by destabilizing NEK2 [30]. However, few studies have reported on another USP7 novel inhibitor P22077. P22077 was reported to induce apoptosis and efficiently inhibit tumor growth USP7-HDM2-p53 axis in neuroblastoma [31]. We got the similar results in HCC cells that P22077 can suppress cell growth. We also observed P22077 treatment could greatly induce dead cells that positive for both Annexin V and PI. The phosphatidylserine (PS) is a lipid that normally restricted to the inner leaflet of the plasma membrane. However during apoptosis, PS exposed on the outer leaflet due to loss of lipid asymmetry. Fluorescently labeled Annexin V can bind to PS and be used to detect PS to measure early and late apoptotic cells. While necrotic cells also show stained with Annexin V due to ruptured membranes that permit Annexin V to access the entire plasma membrane. Therefore, both late apoptotic cells and necrotic cells show positive for Annexin V and PI. Further experiments will be taken to distinguish between late apoptosis or necrosis after P22077 treatment.

Drug resistance is a key factor in poor prognosis for HCC patients. The USP7 inhibitor P5091 has been reported to induce apoptosis of multiple myeloma (MM) cells, and synergistic anti-MM effects can be exerted by multiple drugs. In addition, P5091 inhibits tumor growth and synergizes with other chemotherapeutic agents such as lenalidomide, HDAC inhibitor or dexamethasone to induce synergistic anti-multiple myeloma effect [32, 33].Our data shows P22077 significantly enhances the sensitivity and overcomes resistance of Dox in HCC. Together, we postulate that therapeutic targeting of USP7, using either P22077 or Dox, in patients with HCC may be most effective as an adjuvant therapy.

From the mechanistic aspect, Huh7 cell was treated with P22077 and detected by MS. We found the down-regulated proteins were strongly associated with translational initiation, protein targeting to membrane and viral transcription. These results indicated P22077 restrained HCC proliferation by control the transcription of some oncogenes. In the previous report, P22077 significantly suppressed the growth of human neuroblastoma cell lines which MYCN was amplified [34]. Notably, the up-regulated proteins were involved in signal transduction and cell proliferation. It suggested that P22077 regulates the growth of HCC by various pathway. Obviously, the histone H3 was distinctly decreased, H3K4me^2^ is a methylated form that is involved in the regulation of epigenetics. Western Blot shows H3 and H3K4me^2^ were high expression in SK-Hep1 and Huh7 cells. These results indicated P22077 play an anti-proliferation role in HCC through labializing H3 and H3K4me^2^ with dependent USP7 function. Considerable literature, USP7 was located in nuclear by immunofluorescence, and H3 was a nucleoprotein which usually used be a marker [35]. Histone methylation is an inevitable part of histone modification, including H3K4 monomethylation (H3K4me^1^), H3K4 demethylation (H3K4me^2^), H3K4 trimethylation (H3K4me^3^), H3K9me, H3K27me, H3K36me^3^, H3K79me, and H3K79me^2^ [36–38], which involves a variety of biological processes [39]. Therefore, we suspect that the lethal effect of P22077 on liver cancer cells by inhibiting USP7 deubiquitinating of H3, the specific mechanism remains to be further studied.

In summary, our study is the first to report the potential of USP7 inhibitor P22077 therapy for liver cancer. It has shown that P22077 may hold a great promise as a therapeutic drug to improve the outcome of hepatic carcinoma patients. P22077 is able to inhibit cell proliferation and colony formation in response to the marked inhibition of H3 and H3K4me^2^ in HCC. Given the broad inhibition of P22077 and the antitumor effect in HCC, we suggest P22077 may possess therapeutic potential in the treatment of liver cancer. The above studies indicate that another new direction for the development of such drugs will be the development of synergistic double or even multiple inhibitors, and more deubiquitinases and corresponding inhibitors may be found in the future, making targeted drug therapy for tumors a promising reality.

## Conclusions

We demonstrate a critical role of USP7 in HCC devolvement and chemoresistance. Disruption of USP7 function results in dis-regulated several key biological processes and subsequently activates BAX. USP7 might be a novel and drug-able target in HCC.

## Supplementary information

**Additional file 1.** a HuH7 and SK-Hep1 stable cells were used for 2 weeks. The number of colonies were counted and stained with crystal violet. b Anchor-dependent colony formation was measured after 2 weeks. c Cell viability was measured with CCK-8 assays in SK-Hep1 and Huh7 cells. d The expression of USP7 was detected by Western Blot in HuH7 and SK-Hep1 stable cells. e SK-Hep1 stable cells were prepared and stained with PI and Annexin V. Cells were analyzed using a flow cytometry. The apoptotic percentage: left (UR 1.99, LR 1.78), middle (UR 3.88, LR 4.16), right (UR 3.27, LR 6.69). f The effect of USP7 deletion on cell migration was examined by wound healing assay.

## Data Availability

The datasets used and analyzed in the current study are available from the corresponding author in response to reasonable requests.

## Abbreviations

USP7: ubiquitin-specific protease 7
HCC: hepatocellular carcinoma
UBLs: ubiquitin-like regions (UBLs)
ROC: receiver operating curve
AUCs: area under the curve
CCK-8: cell counting kit-8

## References

[1] Siegel RL, Miller KD, Jemal A (2019). Cancer statistics, 2019. CA Cancer J Clin.

[2] Espey DK, Wu XC, Swan J, Wiggins C, Jim MA, Ward E, Wingo PA, Howe HL, Ries LA, Miller BA (2007). Annual report to the nation on the status of cancer, 1975–2004, featuring cancer in American Indians and Alaska Natives. Cancer.

[3] Le Grazie M, Biagini MR, Tarocchi M, Polvani S, Galli A (2017). Chemotherapy for hepatocellular carcinoma: the present and the future. World J Hepatol.

[4] Huang F, Wang BR, Wang YG (2018). Role of autophagy in tumorigenesis, metastasis, targeted therapy and drug resistance of hepatocellular carcinoma. World J Gastroenterol.

[5] Hu H, Brittain GC, Chang JH, Puebla-Osorio N, Jin J, Zal A, Xiao Y, Cheng X, Chang M, Fu YX (2013). OTUD7B controls non-canonical NF-kappaB activation through deubiquitination of TRAF3. Nature.

[6] Popov N, Wanzel M, Madiredjo M, Zhang D, Beijersbergen R, Bernards R, Moll R, Elledge SJ, Eilers M (2007). The ubiquitin-specific protease USP28 is required for MYC stability. Nat Cell Biol.

[7] Kim RQ, van Dijk WJ, Sixma TK (2016). Structure of USP7 catalytic domain and three Ubl-domains reveals a connector alpha-helix with regulatory role. J Struct Biol.

[8] Bhattacharya S, Chakraborty D, Basu M, Ghosh MK (2018). Emerging insights into HAUSP (USP7) in physiology, cancer and other diseases. Signal Transduct Target Ther.

[9] Hu T, Zhang J, Sha B, Li M, Wang L, Zhang Y, Liu X, Dong Z, Liu Z, Li P (2019). Targeting the overexpressed USP7 inhibits esophageal squamous cell carcinoma cell growth by inducing NOXA-mediated apoptosis. Mol Carcinog.

[10] Yao Y, Zhang Y, Shi M, Sun Y, Chen C, Niu M, Zhang Q, Zeng L, Yao R, Li H (2018). Blockade of deubiquitinase USP7 overcomes bortezomib resistance by suppressing NF-kappaB signaling pathway in multiple myeloma. J Leukoc Biol.

[11] Qin D, Wang W, Lei H, Luo H, Cai H, Tang C, Wu Y, Wang Y, Jin J, Xiao W (2016). CDDO-Me reveals USP7 as a novel target in ovarian cancer cells. Oncotarget.

[12] Tavana O, Sun H, Gu W (2018). Targeting HAUSP in both p53 wildtype and p53-mutant tumors. Cell Cycle.

[13] Bernardi R, Ghia P (2017). Reactivating nuclear PTEN to treat CLL. Oncotarget.

[14] Brenkman AB, de Keizer PL, van den Broek NJ, Jochemsen AG, Burgering BM (2008). Mdm2 induces mono-ubiquitination of FOXO4. PLoS ONE.

[15] Cai JB, Shi GM, Dong ZR, Ke AW, Ma HH, Gao Q, Shen ZZ, Huang XY, Chen H, Yu DD (2015). Ubiquitin-specific protease 7 accelerates p14(ARF) degradation by deubiquitinating thyroid hormone receptor-interacting protein 12 and promotes hepatocellular carcinoma progression. Hepatology.

[16] Liu Y, Zhang Y, Wang S, Dong QZ, Shen Z, Wang W, Tao S, Gu C, Liu J, Xie Y (2017). Prospero-related homeobox 1 drives angiogenesis of hepatocellular carcinoma through selectively activating interleukin-8 expression. Hepatology.

[17] Zhu L, Liu R, Zhang W, Qian S, Wang JH (2015). MicroRNA-205 regulates ubiquitin specific peptidase 7 protein expression in hepatocellular carcinoma cells. Mol Med Rep.

[18] Colland F, Formstecher E, Jacq X, Reverdy C, Planquette C, Conrath S, Trouplin V, Bianchi J, Aushev VN, Camonis J (2009). Small-molecule inhibitor of USP7/HAUSP ubiquitin protease stabilizes and activates p53 in cells. Mol Cancer Ther.

[19] Stolte B, Iniguez AB, Dharia NV, Robichaud AL, Conway AS, Morgan AM, Alexe G, Schauer NJ, Liu X, Bird GH (2018). Genome-scale CRISPR-Cas9 screen identifies druggable dependencies in TP53 wild-type Ewing sarcoma. J Exp Med.

[20] Dar A, Shibata E, Dutta A (2013). Deubiquitination of Tip60 by USP7 determines the activity of the p53-dependent apoptotic pathway. Mol Cell Biol.

[21] Zhang C, Lu J, Zhang QW, Zhao W, Guo JH, Liu SL, Wu YL, Jiang B, Gao FH (2016). USP7 promotes cell proliferation through the stabilization of Ki-67 protein in non-small cell lung cancer cells. Int J Biochem Cell Biol.

[22] Scarabel L, Perrone F, Garziera M, Farra R, Grassi M, Musiani F, Russo Spena C, Salis B, De Stefano L, Toffoli G (2017). Strategies to optimize siRNA delivery to hepatocellular carcinoma cells. Expert Opin Drug Deliv.

[23] Altekruse SF, Henley SJ, Cucinelli JE, McGlynn KA (2014). Changing hepatocellular carcinoma incidence and liver cancer mortality rates in the United States. Am J Gastroenterol.

[24] Kudo M (2018). Systemic therapy for hepatocellular carcinoma: latest advances. Cancers..

[25] Njei B, Rotman Y, Ditah I, Lim JK (2015). Emerging trends in hepatocellular carcinoma incidence and mortality. Hepatology.

[26] Zeng Q, Li Z, Zhao X, Guo L, Yu C, Qin J, Zhang S, Zhang Y, Yang X (2019). Ubiquitinspecific protease 7 promotes osteosarcoma cell metastasis by inducing epithelial mesenchymal transition. Oncol Rep.

[27] Jin Q, Martinez CA, Arcipowski KM, Zhu Y, Gutierrez-Diaz BT, Wang KK, Johnson MR, Volk AG, Wang F, Wu J (2019). USP7 cooperates with NOTCH1 to drive the oncogenic transcriptional program in T-Cell leukemia. Clin Cancer Res.

[28] Su D, Ma S, Shan L, Wang Y, Wang Y, Cao C, Liu B, Yang C, Wang L, Tian S (2018). Ubiquitin-specific protease 7 sustains DNA damage response and promotes cervical carcinogenesis. J Clin Invest.

[29] Wang X, Zhang Q, Wang Y, Zhuang H, Chen B (2018). Clinical Significance of ubiquitin specific protease 7 (USP7) in predicting prognosis of hepatocellular carcinoma and its functional mechanisms. Med Sci Monit.

[30] Franqui-Machin R, Hao M, Bai H, Gu Z, Zhan X, Habelhah H, Jethava Y, Qiu L, Frech I, Tricot G (2018). Destabilizing NEK2 overcomes resistance to proteasome inhibition in multiple myeloma. J Clin Invest.

[31] Fan YH, Cheng J, Vasudevan SA, Dou J, Zhang H, Patel RH, Ma IT, Rojas Y, Zhao Y, Yu Y (2013). USP7 inhibitor P22077 inhibits neuroblastoma growth via inducing p53-mediated apoptosis. Cell Death Dis.

[32] Das DS, Ray A, Das A, Song Y, Tian Z, Oronsky B, Richardson P, Scicinski J, Chauhan D, Anderson KC (2016). A novel hypoxia-selective epigenetic agent RRx-001 triggers apoptosis and overcomes drug resistance in multiple myeloma cells. Leukemia.

[33] Mattern MR, Wu J, Nicholson B (2012). Ubiquitin-based anticancer therapy: carpet bombing with proteasome inhibitors vs surgical strikes with E1, E2, E3, or DUB inhibitors. Biochim Biophys Acta.

[34] Tavana O, Li D, Dai C, Lopez G, Banerjee D, Kon N, Chen C, Califano A, Yamashiro DJ, Sun H (2016). HAUSP deubiquitinates and stabilizes N-Myc in neuroblastoma. Nat Med.

[35] Kim J, Lee Y, Lu X, Song B, Fong KW, Cao Q, Licht JD, Zhao JC, Yu J (2018). Polycomb- and methylation-independent roles of EZH2 as a transcription activator. Cell Rep..

[36] Bernstein BE, Humphrey EL, Erlich RL, Schneider R, Bouman P, Liu JS, Kouzarides T, Schreiber SL (2002). Methylation of histone H3 Lys 4 in coding regions of active genes. Proc Natl Acad Sci USA.

[37] Bannister AJ, Schneider R, Myers FA, Thorne AW, Crane-Robinson C, Kouzarides T (2005). Spatial distribution of di- and tri-methyl lysine 36 of histone H3 at active genes. J Biol Chem.

[38] Mohan M, Herz HM, Takahashi YH, Lin C, Lai KC, Zhang Y, Washburn MP, Florens L, Shilatifard A (2010). Linking H3K79 trimethylation to Wnt signaling through a novel Dot1-containing complex (DotCom). Genes Dev.

[39] Barski A, Cuddapah S, Cui K, Roh TY, Schones DE, Wang Z, Wei G, Chepelev I, Zhao K (2007). High-resolution profiling of histone methylations in the human genome. Cell.

