# Supplementary figures and images for: Ubiquitin-specific protease 7 is a drug-able target that promotes hepatocellular carcinoma and chemoresistance
Source: Cancer Cell Int. 2020 Jan 28;20:28. doi: 10.1186/s12935-020-1109-2 (PMC6986148; doi:10.1186/s12935-020-1109-2)

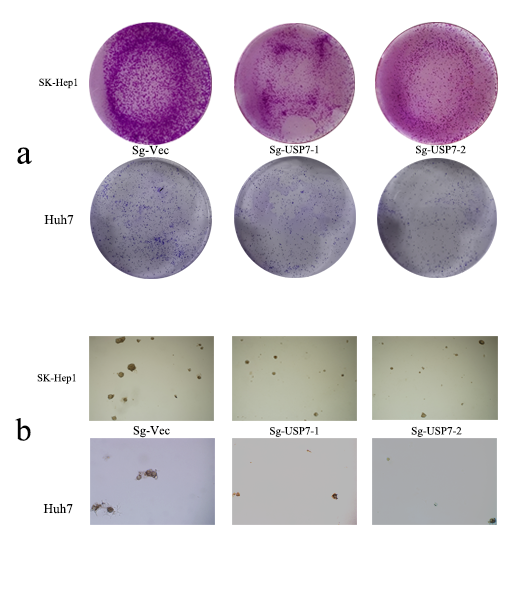

Supplement: Supplementary file 1 — Additional file 1. a HuH7 and SK-Hep1 stable cells were used for 2 weeks. The number of colonies were counted and stained with crystal violet. b Anchor-dependent colony formation was measured after 2 weeks. c Cell viability was measured with CCK-8 assays in SK-Hep1 and Huh7 cells. d The expression of USP7 was detected by Western Blot in HuH7 and SK-Hep1 stable cells. e SK-Hep1 stable cells were prepared and stained with PI and Annexin V. Cells were analyzed using a flow cytometry. The apoptotic percentage: left (UR 1.99, LR 1.78), middle (UR 3.88, LR 4.16), right (UR 3.27, LR 6.69). f The effect of USP7 deletion on cell migration was examined by wound healing assay. [file 12935_2020_1109_MOESM1_ESM.zip › S.FIG-a,b.tif]

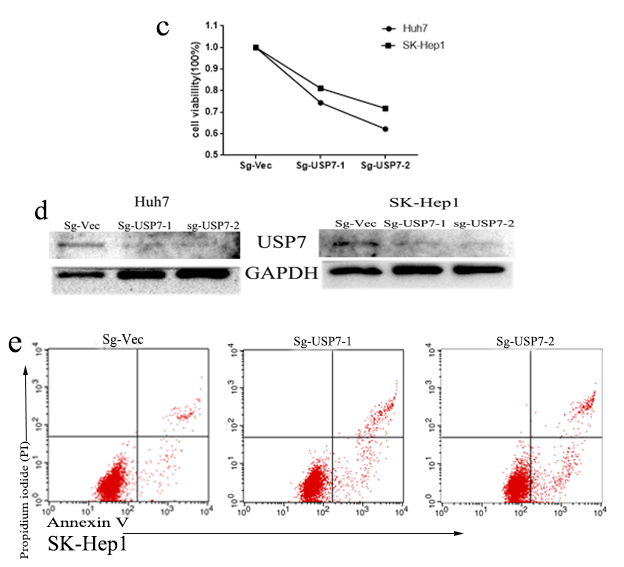

Supplement: Supplementary file 1 — Additional file 1. a HuH7 and SK-Hep1 stable cells were used for 2 weeks. The number of colonies were counted and stained with crystal violet. b Anchor-dependent colony formation was measured after 2 weeks. c Cell viability was measured with CCK-8 assays in SK-Hep1 and Huh7 cells. d The expression of USP7 was detected by Western Blot in HuH7 and SK-Hep1 stable cells. e SK-Hep1 stable cells were prepared and stained with PI and Annexin V. Cells were analyzed using a flow cytometry. The apoptotic percentage: left (UR 1.99, LR 1.78), middle (UR 3.88, LR 4.16), right (UR 3.27, LR 6.69). f The effect of USP7 deletion on cell migration was examined by wound healing assay. [file 12935_2020_1109_MOESM1_ESM.zip › S.FIG-c,d,e.tif]

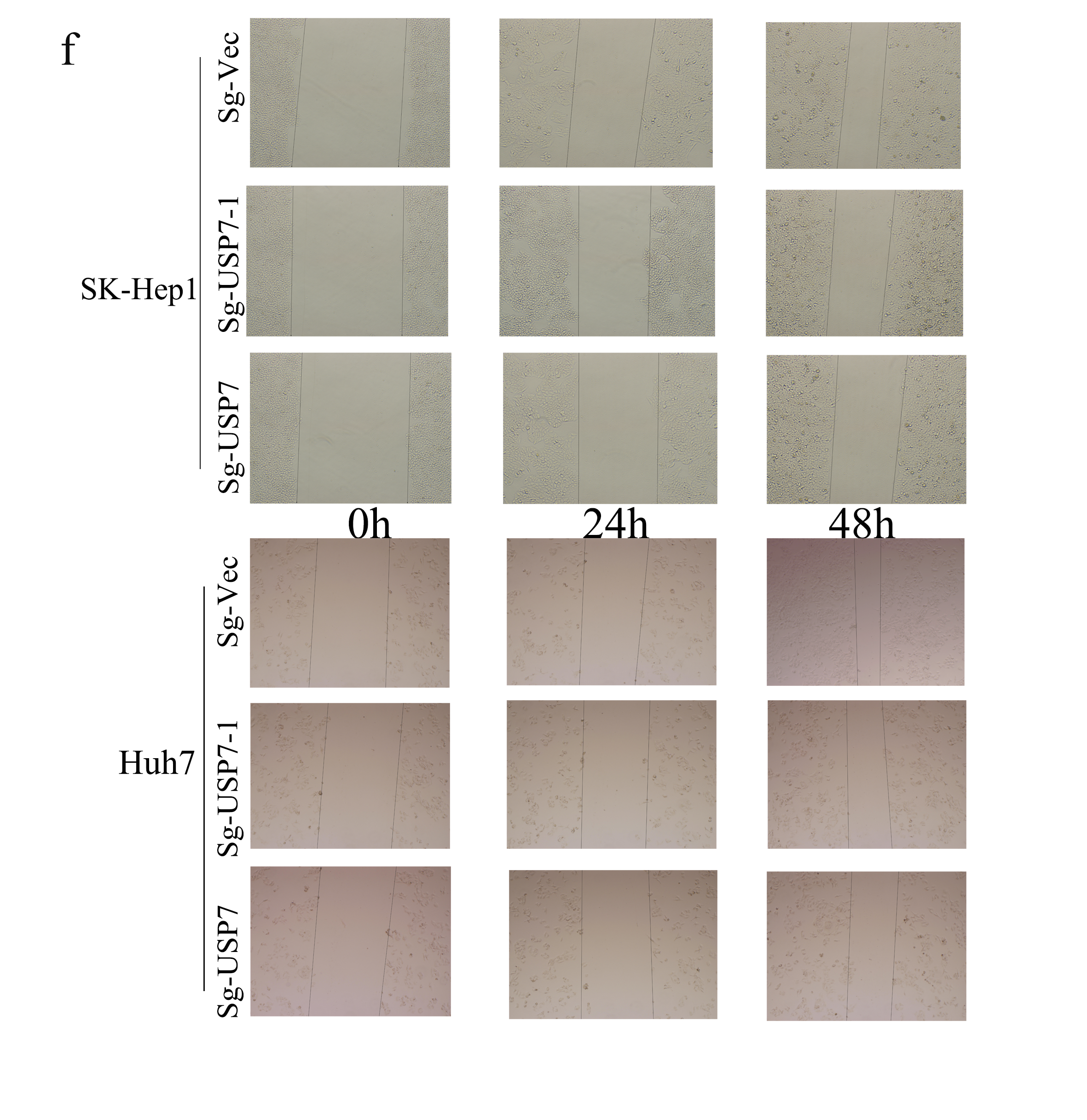

Supplement: Supplementary file 1 — Additional file 1. a HuH7 and SK-Hep1 stable cells were used for 2 weeks. The number of colonies were counted and stained with crystal violet. b Anchor-dependent colony formation was measured after 2 weeks. c Cell viability was measured with CCK-8 assays in SK-Hep1 and Huh7 cells. d The expression of USP7 was detected by Western Blot in HuH7 and SK-Hep1 stable cells. e SK-Hep1 stable cells were prepared and stained with PI and Annexin V. Cells were analyzed using a flow cytometry. The apoptotic percentage: left (UR 1.99, LR 1.78), middle (UR 3.88, LR 4.16), right (UR 3.27, LR 6.69). f The effect of USP7 deletion on cell migration was examined by wound healing assay. [file 12935_2020_1109_MOESM1_ESM.zip › S.FIG-f.tif]
